# Impact of Sub-patent Malaria During Pregnancy on Birth-Weight in Odisha, India: Time-to-Event Analysis of Prospective Longitudinal Follow-Up of a Survey

**DOI:** 10.1007/s44197-022-00082-0

**Published:** 2023-01-17

**Authors:** Madhusmita Bal, Jyoti Ghosal, Arundhuti Das, Sonali Sandeepta, Sanghmitra Pati, Ambarish Dutta, Manoranjan Ranjit

**Affiliations:** 1 Indian Council of Medical Research-Regional Medical Research Centre, Chandrasekharpur, Bhubaneswar, Odisha 751023 India; 2 School of Public Health, KIIT Deemed to Be University, Bhubaneswar, Odisha India; 3 Indian Institute of Public Health, Public Health Foundation of India, Plot No. 267/3408, Jaydev Vihar, Mayfair Lagoon Road, Bhubaneswar, Odisha 751013 India

**Keywords:** Malaria, Pregnancy, Low birth-weight, Sub-patent malaria, India, *Plasmodium falciparum*, *Plasmodium vivax*

## Abstract

**Objective:**

The current study aimed to estimate prevalence of malaria infection, especially sub-patent infection, in pregnant women residing in high malaria-endemic, hard-to-reach pockets of the Indian state of Odisha; and also measure its impact on birth-weight of their new-borns.

**Method:**

A time-to-event analysis of prospective longitudinal follow-up study nested within a cross-sectional survey of people residing in high malaria-endemic six districts of Odisha was conducted during July–November 2019. Malaria status in pregnant mothers was categorized as malaria free; sub-patent, and patent. Hazards Ratio (HR) of low birth-weight (LBW; birth-weight < 2500 gms) was estimated in these three categories (*n* = 308) adjusted for residence (block), gravida, caste, age and gestational age at testing.

**Results:**

50.3% pregnant women had sub-patent malaria infection, 3.9% had patent infection. In fully adjusted model, hazards ratio of LBW was 3.76 (95% CI 1.12, 12.64, *p* = 0.032) in pregnant women with patent infection and 1.82 (95% CI 0.87, 3.81, *p* = 0.109) in women with sub-patent infection when compared to no malaria group.

**Conclusion:**

The study showed that half of the pregnant women in high-endemic pockets had sub-patent infection which posed deleterious influence on birth-weight of their new-borns. The study thereby flags the prevalence of sub-patent infection as a public health concern, because sub-patent infection in pregnant mothers may persist as a “silent” reservoir, with the potential to derail the malaria control program, especially when the country plans malaria elimination by 2030.

## Introduction

Malaria continues to be an international public health problem. Most of the 241 million malaria cases and 62,700 malaria deaths, reported worldwide during 2020, are from Africa and Southeast Asia region. India alone accounted for 83% of estimated malaria cases and about 82% of all malaria deaths in the World Health Organization (WHO) South-East Asia Region [1]. Although every individual living in malaria-endemic areas is at risk of infection, a pregnant woman’s risk of infection is greater due to changes in her hormone levels and immune system [2–4]. Around 60% of pregnant women in the world live in malaria-endemic regions and the prevalence of Malaria in Pregnancy (MiP) ranges from 4 to 40% in Africa to 0.4 to 6.4% in Asia [5–7]. In India the prevalence has been reported to be 0.8–1.3% in Chhattisgarh, 1.8% in Jharkhand and 6.4% in Madhya Pradesh state [5, 8, 9]. MiP when caused by *Plasmodium falciparum (P. falciparum)*, has devastating consequences for both the mother and baby, especially in terms of birth-weight and infant mortality [10, 11].

Malaria can be detected by either microscopic examination of a blood smear or rapid diagnostic test (RDT) or molecular detection assays like polymerase chain reaction (PCR). The sub-patent infection is that infection which is negative for parasites by light microscopy (hereafter, “microscopy-negative infection) or RDT (hereafter, “RDT-negative infection”) but positive for parasites only by PCR-based assay (hereafter, “PCR-positive infection”), the test with highest sensitivity [12, 13]. Sub-patent infection, as the name suggests, implies low parasite density, hence not detectable by microscopy and RDT—the tests commonly employed in malaria control programs to detect malaria case, but only detectable through PCR-based test. However, PCR-based test for malaria has limited role as a routine test in program settings because unlike RDT, it is not a point-of-care test and can only be carried out in specialized laboratories. Therefore, until now, PCR-based assay is only used for the purpose of malaria research. As countries and regions gear up for elimination of malaria by 2030, the role of sub-patent infection assumes critical importance [14]. Persistence of sub-patent infection in the community, who are also largely asymptomatic and remain undetected by conventional tests, may act as malaria reservoirs [6]. Therefore, if left unaddressed, the pool of sub-patent infection can derail elimination campaigns or lead to resurgence after elimination of “patent” malaria [15]. Also, there is scanty evidence regarding the independent impact of sub-patent infection on health of the people affected, particularly pregnant women and their new-borns. Most of the research around sub-patent infection has been focusing on its role as a potential malaria reservoir [16, 17] but not as a risk factor for ill-health. Moreover, the existing evidence about the health effect of sub-patent infection is inconclusive. For example, while the deleterious associations between microscopy and RDT detectable malaria during pregnancy and maternal anemia at delivery and low birth-weight (LBW) is well established [11, 18] but studies on impact of sub-patent infection during pregnancy on these adverse outcomes are not only rare, but also their results are divergent. A study conducted recently in Malawi has shown no association between the sub-patent antenatal *P. falciparum* infection with adverse pregnancy outcomes [13]. Similarly, another study conducted also in Malawi has observed an increased risk of placental malaria but not adverse maternal or fetal outcomes in sub-patent antenatal malaria infection in women during pregnancy [19]. But the reports published from Sudan [20] and Benin [21] have shown associations between sub-patent antenatal *P. falciparum* infection and LBW, preterm delivery. From India, there is hardly any evidence regarding prevalence of sub-patent parasitemia and its health impacts. Realizing the scarcity of evidence in this domain, we set out to examine the burden and effect of malaria among pregnant women in the Indian state of Odisha, so that the Indian malaria program can be informed appropriately. Odisha with 4% of the country’s population contributes to 40% of the malaria cases of the country [22]. Therefore, we aimed to assess the burden of sub-patent (i.e., RDT negative, and PCR-based assay positive) malaria infection during pregnancy in women residing in malaria-endemic, hard-to-reach pockets of the state [22]. We also aimed to analyze the impact of their malaria infection, especially sub-patent infection on birth-weight of their new-borns.

## Materials and Methods

### ‘DAMaN’ Public Health Proramme and Its Assessment

Since 2017, in Odisha, a colossal state-run public health program, namely Durgama Anchalare Malaria Nirakaran (translation: malaria elimination in less accessible areas), in short DAMaN, is being implemented to complement the ongoing malaria control activities of the National Vector-borne Disease Control Programme of India, to accelerate the decline of malaria in the state. DAMaN is being implemented in hard-to-reach pockets of 23 malaria-endemic districts of Odisha. The components of DAMaN mainly included biannual active case detection, enhancement of Long-lasting Insecticidal Net (LLIN) use and strengthening of Indoor Residual Spray (IRS). An assessment of the activities and impact of DAMaN was undertaken in 2019, the details of which have been described elsewhere [22].

### Study Setting and Design

As a part of this assessment exercise, a multi-phase cross-sectional survey was conducted in six DAMaN-implementing districts of Odisha, representing three distinct geophysical regions of the state (Northern plateau: Sundargarh and Keonjhar, Central table land: Anugul and Kandhamal and Eastern ghat (hilly region): Rayagada and Kalahandi). Two DAMaN-implementing districts from each region, one representing higher and the other lower than median Annual Parasite Index (API), the number of confirmed cases of malaria per 1000 population (2015), were selected. Consequently, 1823 households were surveyed between June and November 2019, which is considered as high malaria transmission season in India. These households were selected randomly using a multi-stage clustered sampling technique [22]. Approximately, one out of six sampled households had a woman who was pregnant at the time of survey regardless of any number of past pregnancies, and who consented to provide blood specimen for testing (*n* = 338). Only 12 pregnant women refused to provide specimen. The current study, nested within the DAMaN assessment, was a time-to-event analysis of prospective longitudinal follow-up of pregnant women encountered during the survey [23].

### Outcome Assessments

All enrolled women were longitudinally followed-up until they underwent abortion (medical or spontaneous), experienced a still-birth or delivered a live fetus. Out of 338 enrolled women, 308 delivered live fetuses with validated records of birth-weights of their neonates. Birth-weights were recorded by Auxiliary Nurse and Midwives (ANMs) or Accredited Social Health Activists (ASHAs) immediately after birth using digital weigh scales, reporting weight to the nearest 10 g. LBW was defined as a birth-weight of < 2500 g. The gestational age when blood specimen was collected and when a live fetus was born was estimated using the date of last menstrual period before pregnancy, which was recorded during the survey.

### Laboratory Procedures

During the survey, 1 ml of venous blood specimens was collected in BD Vacutainer^®^ Ethylene Diamine Tetraacetic Acid (EDTA) tubes from these women for parasite detection by RDT and PCR-based assay. RDTs were performed at the point of care using the Pf/PAN RDT (SD Biosensor, India) as per the manufacturer’s recommendation. A positive test result was defined as the presence of the control band and *P. falciparum* and/or other *Plasmodium* infection. Those who tested positive by RDT were referred to the local malaria program for administration of standard anti-malaria treatment. PCR detection of parasites was performed using the species-specific primers (*Plasmodium*. *falciparum*, *Plasmodium. vivax*, *Plasmodium. malariae*, and *Plasmodium*. *ovale*) targeting 18S ribosomal Ribonucleic Acid (rRNA) and cycling parameters as described by Snounou and others [24]. Briefly, genomic Deoxyribonucleic Acid (DNA) was extracted from blood using the QIAamp^®^ DNA Blood Mini Kit (QIAGEN, Germany) and eluted in 50 μl of distilled water. The primary PCR was performed in a 25 μL reaction mixture that contained 0.2 Units of Taq DNA polymerase (GCC Biotech, India), 0.2 mM each Deoxynucleoside triphosphate (dNTP) (HIMEDIA Laboratories, India), 0.4 mM each primer (GCC Biotech, India) and 2.0 mM MgCl2 (GCC Biotech, India). The reaction was allowed to proceed for 35 cycles after an initial denaturation at 94 ℃ for 1 min (min), annealing at 50 ℃ for 1 min, and extension at 72 ℃ for 1 min; final extension was at 72 ℃ for 10 min in a Thermal Cycler (Agilent, USA). The nested PCR reaction condition was the same as primary PCR except for the annealing temperature, 55 ℃. The PCR products were visualized under ultraviolet light following the electrophoresis on 1.2% (w/v) ethidium bromide-stained agarose gel, and images were captured using a gel documentation system (Alpha Image Tech, USA).

### Data Analyses

The demographic characteristics of the sampled respondents were summarized using descriptive statistics. Survival (time-to-event) analysis was carried out employing Cox proportional-hazards regression model [25] to estimate the Hazards Ratio (HR) of LBW among two sub-groups of pregnant women, one with patent malaria infection (both RDT and PCR positive) and the other with sub-patent malaria infection (RDT negative but PCR positive), in comparison to the control group that is those who were infection-free (both RDT and PCR negative). The gestational age at the time of delivery of the live fetus was considered as the time variable and whether the neonate was LBW (Yes/No) as the event variable.

The Cox models were only adjusted for maternal age initially. We a priori hypothesized that remote location, tribal membership (a variable closely related to socio-economic deprivation, residence in high-transmission forest areas and often limited access/utilization of malaria services), gestational age at malaria testing and parity of the pregnant women can confound the malaria–LBW relationship. Therefore, we controlled for these variables (full-adjusted models) to estimate the independent relationship between MiP and LBW. All the statistical analyses were performed in R version 4.1.2.

### Ethical Statement

The ethical approval has been obtained by both the Institute Human Ethical Committee (ICMR-RMRC/IHEC-2019/012 dated 27/02/2019) and State Research & Ethics Committee of Department of Health and Family Welfare, Govt. Of Odisha (453/SHRMU/187/17 Dated 22/8/2017). A signed informed consent was obtained from all the respondents. Respondents consenting to provide their demographic and study related information were only interviewed. Further, 1 ml of their venous blood specimens was collected from the consenting respondents. The details of all the respondents were anonymised at the analysis stage. Moreover, positive malaria test results done at the point of care as well as laboratory were conveyed to the respondents and concerned health officials for necessary action as per program guidelines.

## Results

**Fig. 1:**
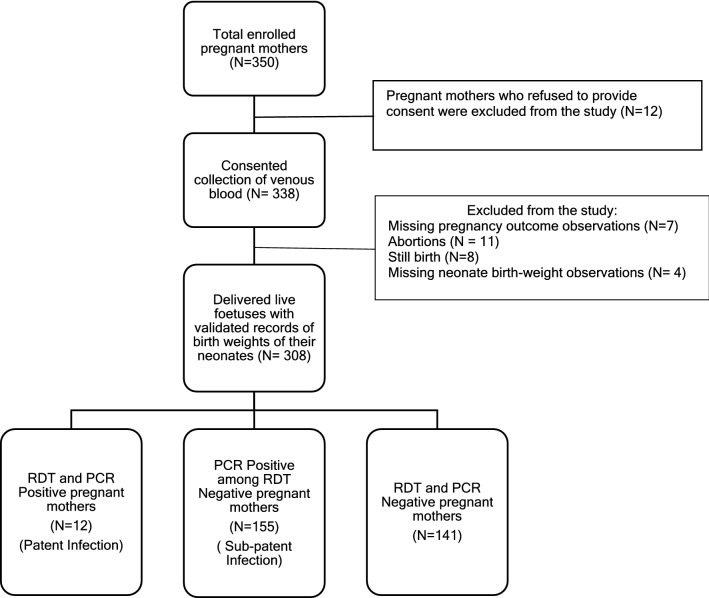
Flow chart illustrating enrolled pregnant women with their malaria test result at the time of survey along with the validated records of their neonates’ birth-weight

The study included 308 pregnant mothers who delivered live fetuses and whose birth-weights were available (Fig. 1). Sixty percent women were multigravida (having experienced pregnancy previously), had a median age of 25 (± 6) years, predominantly belonged to tribal community (74.4%) and only 40.3% of them attained any formal education. Half of the pregnant women in our sample had sub-patent malaria infection followed by 3.9% having patent infection. Their median gestational age at the time of collection of their blood specimens was 170.5 days (Inter-quartile range: 94.2). The median gestational age at which they gave birth was 280 days (Inter-quartile range: 14) (Table 1). Usage of LLIN was very high among these households (98%).

**Table 1:** Demographic characteristics of the study population and prevalence of sub-patent malaria infection

Characteristics	Total (*N* = 308)
Age (in years)	
Median (inter quartile range)	25 (6)
Range	16.0–45.8
Education	
Illiterate	121 (40.3%)
1–8	79 (26.3%)
9–10	71 (23.7%)
11–12	23 (7.7%)
Above 12	6 (2.0%)
Caste	
General	17 (5.5%)
OBC	27 (8.8%)
Scheduled caste	35 (11.4%)
Scheduled tribe	229 (74.4%)
District	
Anugul	94 (30.5%)
Kalahandi	20 (6.5%)
Kandhamal	59 (19.2%)
Keonjhar	35 (11.4%)
Rayagada	27 (8.8%)
Sundargarh	73 (23.7%)
Gestational age at interview (in days)	
Median (IQR)	170.5 (94.2)
Range	32.0–291.0
Gestational age at term (in days)	
Median (IQR)	280 (14)
Range	190–300
Gravida	
Primigravida	120 (39.5%)
Multigravida	184 (60.5%)
Malaria status	
RDT and PCR Negative	141 (45.8%)
PCR Malaria Positive among RDT Negative (Sub-patent infection)	155 (50.3%)
RDT and PCR Malaria Positive (Patent Infection)	12 (3.9%)
LLIN usage	293 (97.7%)

### Association of Malaria Infection in Pregnancy and LBW

The proportion of LBW was found to be 10.6% in malaria-free controls followed by 17.4% in sub-patent malaria infection group and 33.3% in pregnant women with patent infection (the *p *value for unadjusted trend < 0.05).

The age-adjusted hazards of delivering a new-born with LBW was 4.06 (95% Confidence Interval (CI) 1.34, 12.32, *p* = 0.0132) times higher in pregnant women with patent infection followed by 1.74 times (95% CI 0.92, 3.28, *p* = 0.0869) in women with sub-patent infection as compared to the malaria-free control group. The Hazard Ratios (HRs) attenuated to 3.76 (95% CI 1.12, 12.64, *p* = 0.0321) and 1.82 (95% CI 0.87, 3.81, *p* = 0.1099), respectively, in the fully adjusted models (Table 2). The age-adjusted and fully adjusted HRs for LBW in the group with sub-patent infection, although clinically significant, were not statistically significant (both *p* values > 0.05).

**Table 2:** Distribution of LBW in three groups of pregnant women and the Hazards ratio of LBW in patent and sub-patent group in reference to the malaria-free group

Malaria status		Crude *N* (%) of low birth-weight (*p* value for chi-square test)		Age adjusted Hazards Ratio (HR) (95% Confidence Interval), *p* value	Fully adjusted Hazards Ratio (HR)* (95% Confidence Interval), *p* value
	*N* = 308				
RDT & PCR malaria negative	141	15 (10.6%)	*p* = 0.05	Ref	Ref
PCR Positive among RDT negative (sub-patent malaria infection)	155	27 (17.4%)		1.74 (0.92, 3.28), 0.0869	1.82 (0.87, 3.81), 0.1099
Both RDT and PCR positive (patent infection)	12	4 (33.3%)		4.06 (1.34, 12.32), 0.0132	3.76 (1.12, 12.64), 0.0321

*Adjusted with maternal age, caste, geographical location, gestational age at antenatal enrollment, gravida

## Discussion

The current study reveals that more than half of the pregnant women residing in hard-to-reach high-endemic malaria pockets of the Indian state of Odisha were infected with malaria during high-transmission season. Of them, only a small fraction could be detected by RDT, which is routinely used by the malaria control program. However, 50% of all the pregnant women, who returned a negative rapid test, tested positive for malaria by PCR-based assay. According to Rogerson and others (2018), RDT have been poor at detecting malaria in pregnant women, while PCR-based assay was highly efficient in detecting the low-density infection prevalent among pregnant women, [18]. Previous studies from India showed much lesser prevalence of MiP in high-endemic areas when compared to the current study [5, 8, 9]. The higher prevalence of MiP observed in the current study could be because both patent and sub-patent infection were explored in the study, compared to the previous Indian studies, which measured only patent infection. Additionally, the current study was conducted only in areas having high malaria endemicity and during high-transmission season. These study conditions might have differed from those of the previous Indian studies, contributing to the observed higher prevalence. Studies from many countries of Africa and Asia have reported considerable prevalence of symptomatic and asymptomatic malaria including sub-patent infection among pregnant women [6, 26–28]. But, ours is the first study from any region of India, to our knowledge, to report the presence of sizeable magnitude of sub-patent infection in pregnant women. As compared to patent infection, the prevalence of sub-patent infection was much higher in our sample. This provides critical information to the local malaria elimination program, that despite negligible presence of RDT positive infection in pregnant women due to recent spectacular decline in malaria in high-endemic areas of the state [29], the prevalence of sub-patent infection may still be significantly high. This finding poses a few critical questions to NVBDCP of India and the malaria research community in the country as well as globally, especially in the context of elimination of malaria from India by 2030—(a) Does the burden of sub-patent infection we observed reflect the situation in other similar high malaria-endemic areas in the country? (b) If yes, then should a policy be drawn to diagnose sub-patent infection? (c) If yes, how to incorporate diagnosis of sub-patent infection in the program and whom to target? (d) What would be the treatment plan for individuals with sub-patent infection, if any, especially pregnant women and children?

The low-density parasitemia, attributable to immune tolerance due to multiple and often untreated infection with *P. falciparum*, are commonplace in high-endemic areas [30]. Recent research has shown that low-density parasitemia are present in low-endemic regions also, after a period of high malaria transmission [31]. Sub-patent infection mostly do not cause any symptom, and they cannot be detected by routine tests also, hence cannot be readily treated [31]. Many studies have shown that low-density, sub-patent infection can infect mosquitoes leading to malaria transmission. Given that sub-patent infection, are usually not detected through routine investigations, they act like reservoirs and lead to future periods of patent infection [32–34].

Apart from its public health importance as a ‘silent’ reservoir, another likely yet unclear impact of sub-patent infection during pregnancy is its effect on the fetus, evidence regarding which is scanty and nebulous. Some studies reported no clinical impact of sub-patent infection during pregnancy [19, 35], whereas, others have reported higher probability of LBW babies in pregnant women with low-density parasitemia [20, 21]. Against this backdrop, our study illustrated the odds of LBW to be almost four times higher in antenatal mothers with patent infection, a phenomenon already known. But most importantly, what our current study illustrates is a two-fold higher risk of LBW in mothers with sub-patent infection compared to those who were not infected. This makes our study the first to report deleterious impact of sub-patent infection during pregnancy on birth-weight of new-born from India. Our current findings are almost similar to the observations made in eastern and central Sudan [20], Gabon [36] and Kenya [37] but in contrast to that observed in Malawi [38, 39] and Ghana [40].

MiP threatens the well-being of the mother and her developing fetus. An infected mother is likely to be an important reservoir of *Plasmodium* infection. *P. falciparum-*infected erythrocytes express a unique variant surface antigen, Variant Chondroitin Sulfate A (VAR2CSA), which mediates sequestration in the placenta leading to placental infection and inflammation. Sequestration leads to dysregulated expression of cytokines and chemokines, abnormal placental development and reduced placental nutrient transport to fetus, contributing to higher rates of LBW in malaria-infected mothers [26, 30]. But the moot point remains whether parasitemia in low density hampers fetal growth. Our study adds to the body of evidence in this critical area that sub-patent malaria infection during pregnancy contributes to LBW among new-born.

Our study has a few limitations, the primary among them was the lack of statistical significance of the association between sub-patent infection and LBW, although the effect size of the relationship measured using hazard ratio was clinically significant. We calculated post hoc the power of our sample and found it to be only 40% for identifying an effect size of 1.9, the hazard ratio of LBW we found in the sub-patent group. Therefore, the lack of statistical significance probably does not undermine our all-important conclusion that sub-patent infection leads to LBW, because it is most likely due to lack of power (owing to relatively small size) and not due to absence of actual deleterious relationship between sub-patent malaria and birth-weight. Moreover, the dose–response relationship that we observe between malaria and LBW further strengthens the posited causal association between MiP and birth-weight. The second limitation was the ascertainment of malaria infection only once during pregnancy and that too at different gestational sages of the participants. However, it does not undermine our inferences, because malaria-infected women overall showed greater propensity for delivering LBW neonates even after adjustment for the timing of the test. The third limitation of the study is non-use of microscopy for initial parasite detection. The study instead used the RDT method, which is the routine test used in national malaria programs. The strengths of our study include laboratory-based PCR tests of more than 300 pregnant women in a hard-to-reach Indian setting, adjustment with the area (block) variable to control for ecological heterogeneity between regions—a crucial determinant of malaria infection—and lastly adjusting for differences in social determinants, such as education, caste and parity which also have important bearing on birth-weight.

## Conclusion

To conclude, our findings show high prevalence of sub-patent malaria in pregnant women, residing in high-endemic pockets of the Indian state of Odisha. Additionally the study also demonstrates that sub-patent infection has substantial deleterious impact on the birth-weight of their new-borns. The study thereby underlines that sub-patent infection during pregnancy is a major public health concern which requires immediate attention as the country heads towards malaria elimination by 2030. It underscores the need to further (1) evaluate the importance of sub-patent infection as a reservoir for transmission, (2) determine the importance of sub-patent infection during pregnancy in terms of various health outcomes of mother and child, and (3) intensify the research for development of point-of-care molecular diagnostic tools to identify individuals harboring sub-patent infection in the community.

## Data Availability

The data generated or analyzed during the current study are available from the corresponding author on reasonable request.

## Abbreviations

LBW: Low birth-weight
HR: Hazards ratio
RDT: Rapid diagnostic test
NVBDCP: National Vector Borne Disease Control Programme
CDM & PHO: Chief district medical and public health officer
VBDC: Vector-borne disease consultants
VBDTS: Vector-borne disease technical supervisors
CHC: Community Health Centre
PHC: Primary Health Centre
ANM: Auxiliary nurse and midwives
ASHA: Accredited social health activists
AWW: Anganwadi workers
WHO: World Health Organization
MiP: Malaria in pregnancy
*P. falciparum*: *Plasmodium falciparum*
PCR: Polymerase chain reaction
PT: Pre-term
DAMaN: Durgama Anchalare Malaria Nirakaran
API: Annual parasite index
EDTA: Ethylenediaminetetraacetic acid
rRNA: Ribosomal ribonucleic acid
DNA: Deoxyribonucleic acid
μl: Micro-liter
mM: Milli-molar
min: Minutes
dNTP: Deoxynucleoside triphosphate
MgCl_2_: Magnesium chloride
*w*/*v*: Weight/volume
IQR: Inter-quartile range
CI: Confidence interval
VAR2CSA: Variant chondroitin sulfate A
